# The Effect of Ni Addition onto a Cu-Based Ternary Support on the H_2_ Production over Glycerol Steam Reforming Reaction

**DOI:** 10.3390/nano8110931

**Published:** 2018-11-08

**Authors:** Kyriaki Polychronopoulou, Nikolaos Charisiou, Kyriakos Papageridis, Victor Sebastian, Steven Hinder, Aasif Dabbawala, Ayesha AlKhoori, Mark Baker, Maria Goula

**Affiliations:** 1 Department of Mechanical Engineering, Khalifa University of Science and Technology, Main Campus, Abu Dhabi, P.O. Box 127788, UAE; ayesha.alkhoori@ku.ac.ae; 2 Center for Catalysis and Separation, Khalifa University of Science and Technology, Abu Dhabi, P.O. Box 127788, UAE; 3 Laboratory of Alternative Fuels and Environmental Catalysis (LAFEC), Department of Environmental and Pollution Control Engineering, Western Macedonia University of Applied Sciences, 50100 Kozani, Greece; ncharis@teiwm.gr (N.C.); kpapageridis@gmail.com (K.P.); 4 Chemical and Environmental Engineering Department & Nanoscience Institute of Aragon (INA), University of Zaragoza, 50018 Zaragoza, Spain; victorse@unizar.es; 5 Networking Research Center on Bioengineering, Biomaterials and Nanomedicine, CIBER-BBN, 28029 Madrid, Spain; 6 The Surface Analysis Laboratory, Faculty of Engineering and Physical Sciences, University of Surrey, Guildford GU2 4DL, UK; s.hinder@surrey.ac.uk (S.H.); m.baker@surrey.ac.uk (M.B.); 7 Department of Chemical Engineering, Khalifa University of Science and Technology, SAN Campus, P.O. Box 127788, UAE; asifdabbawala123@gmail.com

**Keywords:** Ni supported catalysts, ternary oxides, Sm-Cu-doped CeO_2_, glycerol steam reforming, H_2_ production

## Abstract

In the present study, Ni/Ce-Sm-xCu (x = 5, 7, 10 at.%) catalysts were prepared using microwave radiation coupled with sol-gel and followed by wetness impregnation method for the Ni incorporation. Highly dispersed nanocrystallites of CuO and NiO on the Ce-Sm-Cu support were found. Increase of Cu content seems to facilitate the reducibility of the catalyst according to the H_2_ temperature-programmed reduction (H_2_-TPR). All the catalysts had a variety of weak, medium and strong acid/basic sites that regulate the reaction products. All the catalysts had very high X_C3H8O3_ for the entire temperature (400–750 °C) range; from ≈84% at 400 °C to ≈94% at 750 °C. Ni/Ce-Sm-10Cu catalyst showed the lowest X_C3H8O3_-gas implying the Cu content has a detrimental effect on performance, especially between 450–650 °C. In terms of H_2_ selectivity (S_H2_) and H_2_ yield (Y_H2_), both appeared to vary in the following order: Ni/Ce-Sm-10Cu > Ni/Ce-Sm-7Cu > Ni/Ce-Sm-5Cu, demonstrating the high impact of Cu content. Following stability tests, all the catalysts accumulated high amounts of carbon, following the order Ni/Ce-Sm-5Cu < Ni/Ce-Sm-7Cu < Ni/Ce-Sm-10Cu (52, 65 and 79 wt.%, respectively) based on the thermogravimetric analysis (TGA) studies. Raman studies showed that the incorporation of Cu in the support matrix controls the extent of carbon graphitization deposited during the reaction at hand.

## 1. Introduction

Nowadays, there is broad agreement within the scientific community that human activities related to fossil energy production and consumption are having a major impact on the planet’s climate. The International Energy Agency (IEA) has calculated that a reduction of 70% of CO_2_ emissions by the year 2060, relative to the 2014 levels, will be necessary, if the rise in global temperatures is not to exceed 2 °C (the so called 2DS scenario) [1]. Although Renewable Energy Systems (RES) are rapidly expanding their foothold on the world’s electricity markets, the transportation sector (accounting for 23% of total CO_2_ emissions) remains heavily reliant on petro-based sources (over 96%) [2]. According to IEA, the share of biofuels (bioethanol and biodiesel) will need to increase to approximately 30% of total fuel consumption by 2060, if the 2DS scenario is to be achieved [3].

Biodiesel is being produced through the transesterification reaction and, although researchers have identified a number of technical barriers that need to be overcome for the future growth of the industry [4], an issue that has remained under the radar is that of the co-production of glycerol (10 wt.% of the oil undergoing the reaction) [5]. The current practice of glycerol incineration in not only environmentally unsustainable, but also underutilizes this resource, which makes the development of innovative solutions a pressing concern. One option that is been explored by the scientific community is its energetic utilization via steam reforming (GSR) for the production of hydrogen or synthesis gas (syngas), both raw materials that have a variety of uses in the petro-chemical industry. 

The glycerol steam reforming reaction (Equation (1)), in essence a combination of glycerol decomposition (Equation (2)) and the water-gas shift reaction (WGS, Equation (3)), shows that 1 mol of glycerol can be converted into 7 mol of hydrogen, however, parallel reactions (such as methanation and carbon formation reactions) can affect this outcome [6,7]. A number of intermediates and by-products may also be formed, further complicating the process [8,9]. The thermodynamic studies that have been undertaken for the GSR conclude that hydrogen production is favored at atmospheric pressure, high temperature and high water to glycerol feed ratio (WGFR) [10,11].

C_3_H_8_O_3_(g) + 3H_2_O(g) → 3CO_2_(g) + 7H_2_(g), (1)

C_3_H_8_O_3_(g) → 3CO(g) + 4H_2_(g) , (2)

CO(g) + H_2_O(g) ↔ CO_2_(g) + H_2_(g), (3)

Obviously, the catalyst plays a key role in the determination of the reaction pathway and product distribution, due to the fundamental steps involved in the cleavage of C–C, O–H and C–H bonds of the molecule of glycerol, and the need to maintain the C–O bonds [12,13]. As a result, a number of works found in the literature report on monometallic systems that use either noble (Pt, Pd, Rh, Ru, Ir) or transition metals (Ni, Co, Cu), with Ni-based catalysts the most investigated thus far e.g., [14,15,16,17,18,19,20,21,22,23,24,25,26]. This is because nickel combines wide availability (thus, it is relatively cheap), good activity for the cleavage of C–C, O–H and C–H bonds, and an ability to catalyze the WGS reaction; the major drawback identified is catalyst deactivation due to carbon formation and metal particle sintering [27,28,29]. 

In a previous work carried out by our group [30], we compared the performance of Ni, Co and Cu catalysts supported on bare alumina between 400 and 750 °C using a Water to Glycerol Feed Ration (WGFR) of 20:1, molar. We also conducted stability tests at 600 °C with a WGFR of 9:1 for 20 h. Although the Ni/Al catalyst had an improved ability to convert glycerol into gaseous products and produce hydrogen, the time-on-stream testing showed that it also deactivated quite drastically. This was in contrast with the behavior of the Co/Al and Cu/Al catalysts, which deactivated with a much slower rate despite the fact that temperature programmed oxidation (TPO) and thermogravimetric analysis (TGA) showed that the latter samples contained more carbon than the Ni/Al. 

The bimetallic systems that have been tested in the GSR usually combine Ni with another metal such as Co, Sn, Pt, Ir, Pd and Ru e.g., [31,32,33,34,35,36]; only a handful of works have investigated the performance of Ni-Cu catalytic systems [34,35,36]. Dou et al. [34,35] compared the performance of Ni-Cu/Al_2_O_3_ (≈29 wt.% Ni, 31 wt.% Cu), Ni-Cu/Mg (≈39 wt.% Ni, 41 wt.% Cu) and Ni/MgO (≈65 wt.% Ni) catalysts in the temperature range of 450 to 650 °C. They reported that at 650 °C, the Ni-Cu/Al_2_O_3_ catalyst had the highest total glycerol conversion (91%) and H_2_ selectivity (93%) and the lowest amount of deposited carbon. Ramesh et al. [36] synthesized copper decorated perovskite catalysts (LaNiO_3_, NaNi_0.9_Cu_0.1_O_3_ and LaNi_0.5_Cu_0.5_O_3_) under vapor phase reaction conditions and carried out 24 h time-on-stream tests at 650 °C, using a WGFR of approximately 9:1. The authors reported that Cu decoration resulted in higher reduction of active Ni species with moderate basicity and although all catalysts were quite stable, the best performance was achieved by LaNi_0.9_Cu_0.1_O_3_, with 73% glycerol conversion and H_2_ selectivity of 67%. Carbon analysis on the spent samples showed that the lowest deposits were found on the LaNi_0.9_Cu_0.1_O_3_, however, the increase in copper concentration led to increased carbon deposition due to the formation of Ni-Cu alloys. Moreover, decoration by copper also seemed to result in the formation of less graphitic carbon allotropes. 

The method of preparation of a catalyst [37,38], the constituent elements and their ratio [39], as well as the complexing agent [40,41] used are of paramount importance for the viability of the catalyst. Ceria, due to its basic character, redox properties and capacity to store lattice oxygen, has the ability to promote water dissociation, methane reforming, and the WGS reaction, as well as minimize the deposition of carbon [42,43,44,45]. In the GSR, ceria has been tested both as supporting and as promoting material with encouraging results [45,46,47]; however, it has never before been tested in conjunction with copper. Liu et al. [48], working on the steam reforming of methanol, reported that the use of ceria led to high copper dispersion, as well as, possible stabilization of Cu^+^ state as a consequence of strong copper–ceria interaction. Furthermore, the addition of samarium oxide (Sm_2_O_3_) into supports has been shown to improve performance in several catalytic reactions, which was ascribed to the excellent thermal stability and good release/storage oxygen capacity of Sm_2_O_3_ [49,50]. To the best of our knowledge, samarium has never been tested as supporting material in the GSR.

Given the background discussed above, we prepared nickel catalysts (8 wt.%) that were supported on Cu (loadings 5, 7, 10 at.%)-CeO_2_-Sm_2_O_3_ (Ni/Ce-Sm-xCu), and tested catalytic activity and time on stream stability for the glycerol steam reforming reaction. Activity tests were carried out between 400–750 °C, while catalyst stability and carbon deposition were examined at 650 °C for 8h. The catalysts were prepared by the wet impregnation technique, while the supports were synthesized by the microwave sol-gel method. To the best of our knowledge, this is the first time that these materials or this particular support preparation method have been used in the GSR. To help achieve our goal, the catalysts’ surface and bulk properties were determined by applying several characterization techniques (Brunauer-Emmet-Teller (BET), X-ray diffraction (XRD), Temperature Programmed Desorption (TPD), temperature-programmed reduction (TPR), x-ray photoelectron spectroscopy (XPS), scanning electron microscope (SEM), Transmission Electron microscopy (TEM)), while carbon deposition was studied using TGA and Raman. The catalytic performance was studied in order to investigate the effect of the reaction temperature on: (i) Glycerol total conversion, (ii) Glycerol conversion to gaseous products, (iii) Hydrogen selectivity and yield, (iv) Selectivity of gaseous products, (v) Selectivity of liquid products, and (vi) Molar ratio of H_2_/CO and CO/CO_2_ in the gaseous products mixture. Notably, quantitative results of the liquid products are reported.

## 2. Materials and Methods

### 2.1. Catalyst Preparation

Supports with low (5 at.%), medium (7 at.%) and high (10 at.%) Cu content were synthesized by a microwave sol-gel method, while keeping the Ce/Sm ratio of 1. The precursor salts, Ce(NO_3_)_3_.6H_2_O (99.95%), Sm(NO_3_)_3_·6H_2_O (99.95%) and Cu(NO_3_)·9H_2_O (99.95%), all Sigma-Aldrich (St. Louis, MO, USA), were dissolved with the appropriate molar ratios in distilled water. The total metal loading [Ce + Sm + Cu] was kept at 0.03 mol in all cases. Citric acid (the complexing agent) was dissolved in distilled water, maintaining the total metal loading to citric acid ratio (Mtot/citric acid) at 0.75, yielding an excess of citric acid. The metal salt and citric acid solutions were then mixed and the final solution was subjected to microwave heating (130 °C/800W) and stirring until a yellowish gel was formed, as explained in recent works by Polychronopoulou et al. [51,52]. Following microwave heating, all synthesized materials were calcined at 500 °C for 6 h under atmospheric conditions to form the mixed oxide catalyst. The experimental set up is described in detail elsewhere [51].

The catalytic samples were prepared using the wet impregnation technique, by impregnating the Ce-Sm-5Cu, Ce-Sm-7Cu and Ce-Sm-10Cu with Ni(NO_3_)_2_.6H_2_O aqueous solution (Sigma Aldrich, St. Louis, MO, USA) having the appropriate concentration (C = 0.17 M), in order to obtain a nominal loading of 8 wt.% Ni in the final catalysts. All slurries were evaporated using a rotary evaporator at 70 °C for 5 h and air dried at 120 °C for 12 h followed by calcination at 800 °C for 4 h. The samples were labeled as Ni/Ce-Sm-5Cu, Ni/Ce-Sm-7Cu and Ni/Ce-Sm-10Cu. 

### 2.2. Catalyst Characterization

The powder X-ray diffraction (XRD) patterns were acquired at room temperature using a Bruker D2 Phaser powder (Bruker, Billerica, MA, USA) diffraction system with Cu-K_α1_ radiation (1.5418Å) operated at 30 kV and 20 mA. For the calculation of the average crystallite size the broadening of the (111) peak using the Scherrer equation was adopted. Cubic indexation method was used for the estimation of the lattice parameter, based on the predominant (111) peak. 

The multi-point Brunauer-Emmet-Teller method, in the relative pressure range 0.05 < P/P_0_ < 0.20, was used to calculate the total specific surface area (SSA). The pore size distribution (PSD) was estimated by the Barrett-Joyner-Halenda (BJH) method. Ultra-high purity (99.9999%) N_2_ was used for the acquisition of adsorption/desorption isotherms at −196 °C using the 3Flex accelerated surface area and porosimetry analyzer (Micromeritics Instruments Corporation, Norcross, GA, USA). The analyzer is equipped with a high-vacuum system, and three 0.1 Torr pressure transducers. Prior to testing, the samples (~120 mg) were degassed under vacuum (10–6 mbar) at 250 °C for 12 h. 

The redox properties of the catalysts were studied using H_2_ temperature-programmed reduction (H_2_-TPR) studies. The experiments were performed using an Autochem 2920, (Micromeritics Instruments Corporation, Norcross, GA, USA), where a 10 vol.%/H_2_/Ar gas mixture (30 NmL/min) flow over ∼0.2 g of the pre-calcined (20 vol.% O_2_/He, 500 °C, 2 h) catalyst was used. A 30 °C /min temperature ramp was adopted, while the Thermal Conductivity Detector (TCD) signal was recorded continuously. The experiments were performed after mounting the catalysts on a U-shaped quartz tube plugged with quartz wool. 

CO_2_-TPD and NH_3_-TPD experiments were conducted using Autochem 2920 (Micromeritics Instruments Corporation, Norcross, GA, USA). In particular, a gas mixture (30 NmL/min) of 5 vol.% CO_2_/Ar and 1 vol.% NH_3_/He respectively, was passed over ~0.15 g of the pre-calcined (20 vol.% O_2_/He, 500 °C, 2 h) catalyst using a temperature ramp of 30 °C /min, while the TCD signal was recorded continuously. The mass numbers (m/z) 15, 30, 44 and 46 were used for NH_3_, NO, N_2_O and NO_2_ during NH_3_-TPDs, while the (m/z) 28 and 44 were used for CO and CO_2_, respectively, during CO_2_-TPDs. 

XPS studies were performed using a ThermoFisher Scientific (East Grinstead, UK) K-Alpha^+^ spectrometer. XPS spectra were collected using a monochromated Al Kα X-ray source (hν = 1486.6 eV). An X-ray spot of ~400 μm radius was employed. A Pass Energy of 200 eV was employed to acquire the Survey spectra, whereas for the high resolution, for core level spectra for all elements, a Pass Energy of 50 eV was used. C1s peak at 285 eV was used as an internal charge reference to correct for charging effects during acquisition. Quantitative surface chemical analyses was implemented using the high resolution, core level spectra following the removal of a non-linear (Shirley) background. The manufacturers Avantage software with the appropriate sensitivity factors and corrections for the electron energy analyser transmission function, was used.

The catalyst morphology was examined with a JSM 7610F-Field Emission Scanning Electron Microscope (JEOL Ltd., Tokyo, Japan), using both secondary electron imaging and low-angle-backscattered imaging. Qualitative energy dispersive X-ray spectroscopy (EDS) was conducted with an Oxford XMax^N^ 50 mm^2^ silicon drift detector that was coupled with the SEM equipment, and an AZtecEnergy analysis software (Oxford Instruments, Abingdon, UK). 

For electron microscopy observation, the catalysts were dispersed in milli-Q water to prepare the samples. After 60 s in an ultrasonic bath, a drop of this suspension was applied to a copper grid (formvar-200 mesh) coated with carbon film, and allowed to dry in air. Electron microscopy images were recorded on a T20-FEI Tecnai thermoionic microscope (ThermoFisher Scientific, Waltham, MA, USA) operated at an acceleration voltage of 200 kV with a LaB6 electron source fitted with a “SuperTwin^®^” objective lens allowing a point-to-point resolution of 2.4 Å.

The amount of carbon deposited on the catalysts was measured with a thermogravimetric analyzer (TGA), on a Leco TGA701 instrument (LECO Corporation, St. Joseph, MI, USA). The thermal decomposition process of the coke formed onto the spent catalysts was also obtained. In the procedure, ≈50 mg of the spent catalyst was subjected to a TGA scan from room temperature (RT) to 1000 °C at a heating rate of 10 °C min^−1^ under a flow of dry air (3.5 L min^−1^). Curie point standards were utilized for the temperature calibration.

The coke deposited on the spent catalyst samples was also characterized by means of Raman spectroscopy. Spectra were collected using a Thermo Scientific DXR Raman Microscope (ThermoFisher Scientific, Waltham, MA, USA) with an excitation wavelength of 532 nm, 100 mW diode laser as the excitation source and laser intensity of ∼5mW. For each sample, at least three Raman spectra were collected in different areas to assess the homogeneity of the investigated material.

### 2.3 Catalytic Tests

The glycerol steam reforming reaction was carried out at atmospheric pressure, in a continuous flow, fixed-bed, single pass, tubular stainless steel reactor, with an inner diameter of 14 mm. The experimental set up used allowed the feeding of both liquid and gaseous streams as it contained two vaporizers, a pre-heater before the reactor and a condenser after it. The vaporizers, pre-heater and reactor were placed into electrical ovens and regulated with programmed-temperature controllers. To prevent overpressure phenomena, pressure controllers were placed before and after the inlet and outlet gas, respectively.

The gaseous products were analyzed on-line by an Agilent 7890A gas chromatograph (Agilent Technologies Inc, Santa Clara, CA, USA), with two columns in parallel, HP-Plot-Q (19095-Q04, 30 m length, 0.530 mm I.D.) and HP-Molesieve (19095P-MSO, 30 m length, 0.530 mm I.D.), equipped with TCD and FID detectors. Liquid products were analyzed via a combination of Gas Chromatography and Mass Spectroscopy. The instrument used was a 7890A/5975C Triple -Axis Detector diffusion pump-based GC-MS equipped with split/splitless inlet (Agilent Technologies Inc, Santa Clara, CA, USA). Chromatographic separation was achieved by a 30 m × 250 µm HP-5MS (5% phenyl, 95% methylpolysiloxane) capillary column with film thickness of 0.25 μm. Helium 5.0 (99.999%) was used as carrier gas at 1 mL min^−1^ in a constant flow rate mode. Detailed information of the instrument calibration and analysis procedure is provided in the supporting information file. 

Prior to catalytic testing, 300 mg of undiluted catalyst (the catalyst bed was supported by quartz wool) was reduced in situ under a flow of 200 mL min^−1^ H_2_ (5.0) at 800 °C for 1 h. The catalyst was then purged with He (5.0) for 45 min and the temperature was lowered to the required level, in accordance with the experimental protocol that was to be followed, and the reaction feed was introduced into the catalyst bed. Two different experimental protocols were followed. 

Experimental protocol #1 was designed with the purpose of investigating catalytic activity and selectivity. For this reason, tests were carried out in the temperature range 400–750 °C. Following the reduction procedure (at 800 °C), the temperature was reduced at 750 °C under a flow of He and then the feed was introduced in the reactor and the first measurement was obtained. The temperature was subsequently reduced by 50 °C steps (down to 400 °C). In order to ensure operation at steady state conditions, the catalyst was left for approximately 50 min at each step. This timeframe allowed us to obtain three measurements at each step regarding the gaseous products. Liquid products were obtained at the end of this 50 min period.

Experimental protocol #2 aimed at investigating the catalytic stability and carbon deposition. For this reason, the catalysts were tested for 8 h at 650 °C, using a fresh sample for every test. The procedure followed was similar to that described above, i.e., activation was done at 800 °C, the catalyst was purged with He, the temperature was reduced accordingly, and the feed was introduced into the reactor. For gaseous products, measurements were obtained at hourly intervals; for liquid products, measurements were taken at the beginning of the test, the middle (after 4 h) and at the end. 

For both experimental protocols, the reaction feed consisted of the liquid stream—an aqueous solution of 20:80 wt.% C_3_H_8_O_3_ and H_2_O (20:1 steam/glycerol molar ratio), with a total liquid flow rate of 0.15 mL min^−1^, which was kept under continuous stirring at room temperature; and the gas stream (Helium 5.0, 91 mL min^−1^). The glycerol used had 99.5% purity and was obtained from Sigma Aldrich (St. Louis, MO, USA). The glycerol/water mixture was fed with a high-performance liquid chromatography pump (Series I) into the evaporator and was vaporized at 350 °C before it was mixed with He. Thus, the gas feed at the reactor’s inlet consisted of a gas mixture of 73% H_2_O, 4% glycerol and 23% He, corresponding to a Weight Hourly Space Velocity (WHSV) of 50,000 mL g^−1^ h^−1^.

### 2.4. Reaction Metrics

Catalytic performance is reported in terms of H_2_ yield, H_2_, CO, CH_4_ and CO_2_ selectivity, glycerol conversion into gaseous products, and total glycerol conversion. Moreover, the performance of the catalysts for the liquid phase products is reported in terms of acetol (C_3_H_6_O_2_), acetone [(CH_3_)_2_CO], allyl alcohol (CH_2_=CHCH_2_OH), acetaldehyde (C_2_H_4_O), acetic acid (C_2_H_4_O) and acrolein (C_3_H_4_O) selectivity. Performance parameters were calculated based on Equations (4)–(9):

%glycerol conversion_(total conversion)_ = ((Glycerol_in_ − Glycerol_out_)/glycerol_in_) × 100, (4)

%glycerol conversion_(gaseous products)_ = (C atoms in the gas products/total C atoms in the feedstock), (5)

H_2_ yield = (H_2_ mol produced/mol of glycerol in the feedstock), (6)

%H_2_ selectivity = (H_2_ mol produced/C atoms produced in the gas phase) × (1/RR) × 100, (7)

% selectivity of *i* = (C atoms in species *i*/C atoms produced in the gas phase) × 100, (8)

% selectivity of *i*’ = (C atoms in species *i*’/C atoms produced in the liquid phase) × 100, (9)

where RR is the reforming ratio (7/3), defined as the ratio of mol of H_2_ to CO_2_ formed; species *i* refers to CO, CO_2_ and CH_4_; and species *i*’ refers to acetol, acetone, allyl alcohol, acetaldehyde, acetic acid, and acrolein.

## 3. Results and Discussion

### 3.1. Characterization Results

#### 3.1.1. Microstructural Characterization

**Figure 1:**
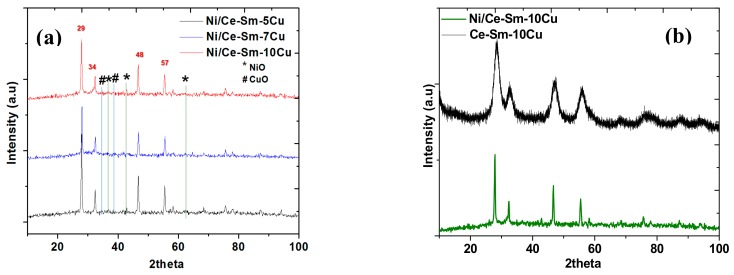
X-ray diffraction (XRD) patterns of the: (**a**) Ni/Ce-Sm-xCu (x = 5, 7, and 10 at.%) catalysts; (**b**) Ni/Ce-Sm-10Cu catalyst and Ce-Sm-10Cu support.

Figure 1 presents the XRD diffraction patterns of the Ni supported catalysts (Figure 1a) along with the Ce-Sm-10Cu for benchmarking (Figure 1b). The patterns correspond to catalysts following their preparation and calcination at 800 °C. The polycrystalline structure of all the catalysts is clearly illustrated by the different sharp diffraction peaks. The typical ceria fluorite structure was identified based on the observed diffraction peaks (i.e., at 29° for plane (111), at 34° for plane (200), at 48° for plane (220) and at 57° for plane (311)). A small peak, characteristic of NiO (JCPDS 34-0394) can be observed at 37.2° and 43.1°, whereas traces of the CuO phase were also observed as indicated by the peaks at 35.4° and 38.6° corresponding to the (002) and (111) planes of CuO phase (JCPDS 05-0661). The extremely weak peaks associated with the CuO and NiO phases corroborate with highly dispersed CuO and NiO nanoparticles over the cubic ternary support or the formation of a interstitial/substitutional solid solution. Adopting the Scherrer formula, the crystallite size of the Ni/Ce-Sm-xCu catalysts was calculated and it was found to be around 63 nm, in agreement with the XRD pattern which is composed of narrow sharp high intensity peaks (Table 1).

In Table 1, the unit cell parameter of CeO_2_ cubic lattice was calculated. The undoped CeO_2_ prepared with the same method of synthesis showed a lattice constant of 5.26 Å [51]. Doping of CeO_2_ with Sm^3+^ cations (following the same preparation method) led to expansion of the lattice constant to 5.29 Å [51], as expected due to the larger radius of Sm^3+^ cation (107 pm) compared to the Ce^4+^ (97 pm). Simultaneous doping with Sm^3+^ and Cu^2+^ gave an ultimate lattice constant of 5.38, 5.40 and 5.42 Å for the Ce-Sm-5Cu, Ce-Sm-7Cu and Ce-Sm-10Cu, respectively. 

**Figure 2:**
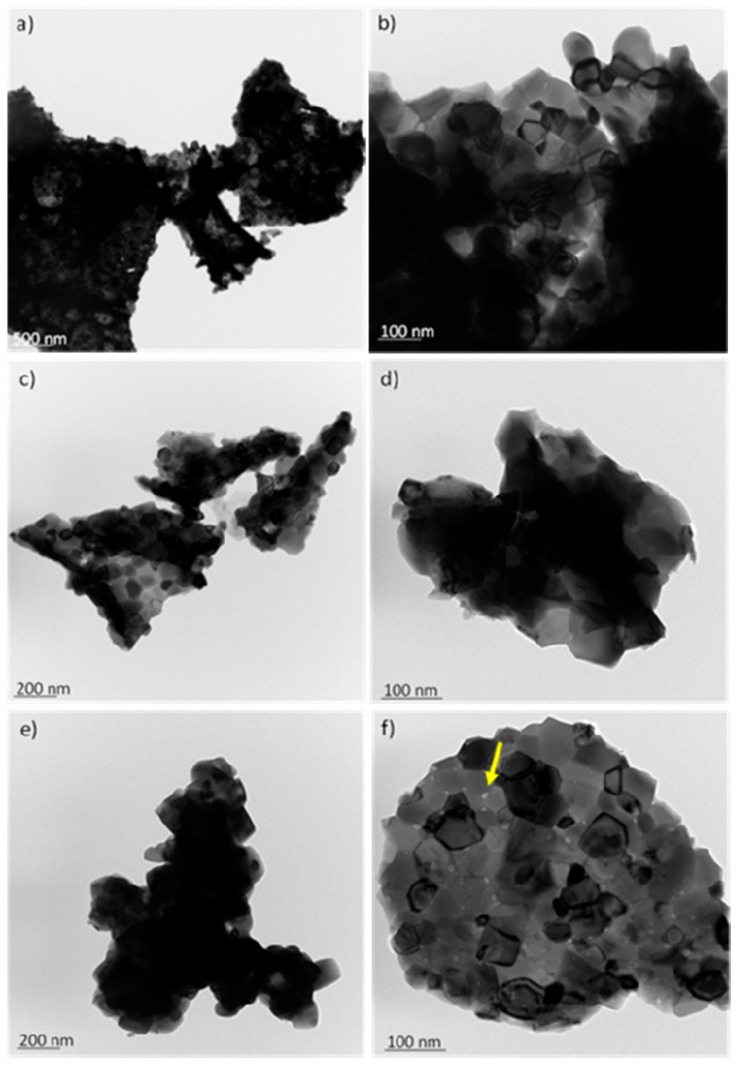
Transmission electron microscopy images with different magnifications of Ni nanoparticles supported on Ce-Sm-xCu catalysts: (**a,b**) 5% Cu; (**c,d**) 7% Cu; (**e,f**) 10% Cu.

This seems to be contradictory if the scenario of a substitutional solid solution of Cu in the Ce-Sm-O cubic lattice is adopted given the smaller size of the Cu^2+^ cations (0.73 Å). It is likely that Cu has been introduced interstitially into the Ce-Sm-O cubic lattice, in agreement with previous work [53]. Addition of the Ni^2+^ cation (0.72 Å) caused an additional small expansion corroborating for the interstitial addition of Ni^2+^ cations. The addition of alio-valent cations such as Ni^2+^, Cu^2+^ and Sm^3+^ is expected to cause strain in the lattice and create defects including oxygen vacancies, which are of crucial importance for the steam reforming reactions [54,55,56,57]. TEM images obtained over the fresh Ni/Ce-Sm-5Cu, Ni/Ce-Sm-7Cu and Ni/Ce-Sm-10Cu catalysts revealed that the catalysts are composed of large particles (mostly non spherical ones). Crystallites in the range of <15 nm can be seen most likely assigned to Ni (Figure 2). More in detail studies are ongoing for the clarification of this aspect.

#### 3.1.2. Textural and Morphological Studies

The N_2_ adsorption-desorption isotherm obtained at −196 °C for the Ni catalysts was used for porosity studies, whereas the pore size and the BET specific surface areas (m^2^/g) are listed in Table 1. Typical IV isotherms were observed (not shown herein) to be in agreement with the International Union of Pure and Applied Chemistry (IUPAC) classification [58]. The N_2_ physical adsorption can be described by low N_2_ volumes (i.e., <5 cm^3^/g) in the low P/P_0_ regime (i.e., 6 × 10^−3^). A small hysteresis loop can be seen between adsorption and desorption in the P/P_0_ range of ~0.5–~0.9. The hysteresis can be associated with capillary condensation of N_2_ gas inside the mesopores [59]. The N_2_ isotherm did not show any plateau at the maximum P/P_0_ point of ~0.99, but it extended in an almost vertical direction. This behavior is typical of macropores (i.e., pore sizes above 50 nm) [58]. Comparing the BET surface area of the supports with the ones herein, it can be stated that Ni impregnation led to a dramatic decrease of the specific surface area (Table 1). 

**Figure 3:**
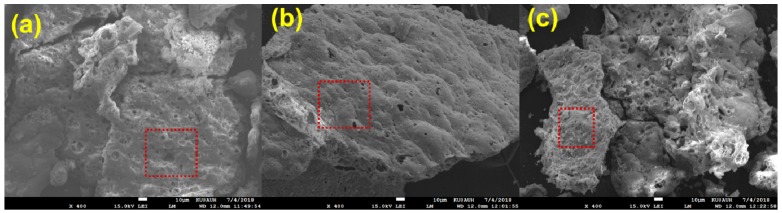
Scanning electron microscopy images of the Ni/Ce-Sm-xCu catalysts: (**a**) 5% Cu; (**b**) 7% Cu; (**c**) 10% Cu.

**Figure 4:**
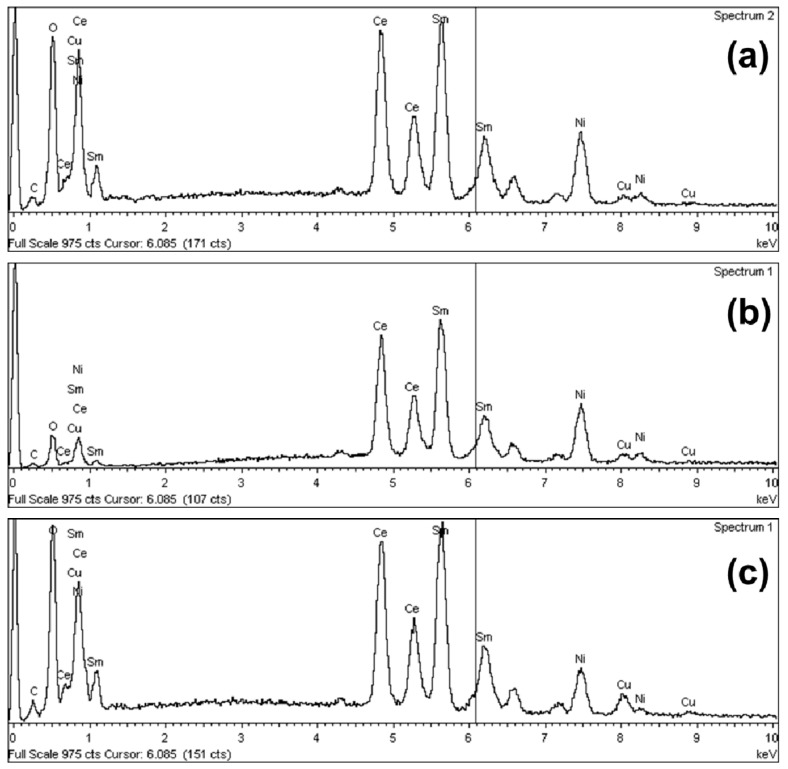
EDX spectra of the Ni/Ce-Sm-xCu catalysts: (**a**) 5% Cu; (**b**) 7% Cu; (**c**) 10% Cu.

The SEM microphotographs of the catalysts are presented in Figure 3 and the corresponding EDX spectra in Figure 4. The Ni catalysts preserve the spongy morphology of the supports [51], so it can be said that the wet impregnation does not change the morphology dramatically. The large voids shown on the microphotographs are due to the gases released during the calcination of the catalysts up to 500 °C. The morphological features of the catalysts are in agreement with the low specific surface area, as presented above, and the N_2_ adsorption-desorption isotherms.

#### 3.1.3. Surface and Redox Properties

**Figure 5:**
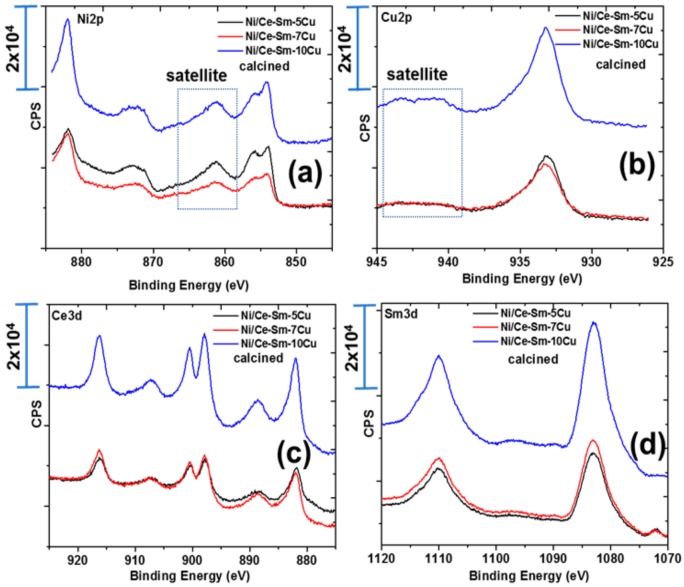
Core level spectra of: (**a**) Ni2p, (**b**) Cu2p; (**c**) Ce and (**d**) Sm3d, respectively, of the calcined Ni/Ce-Sm-xCu catalysts.

**Figure 6:**
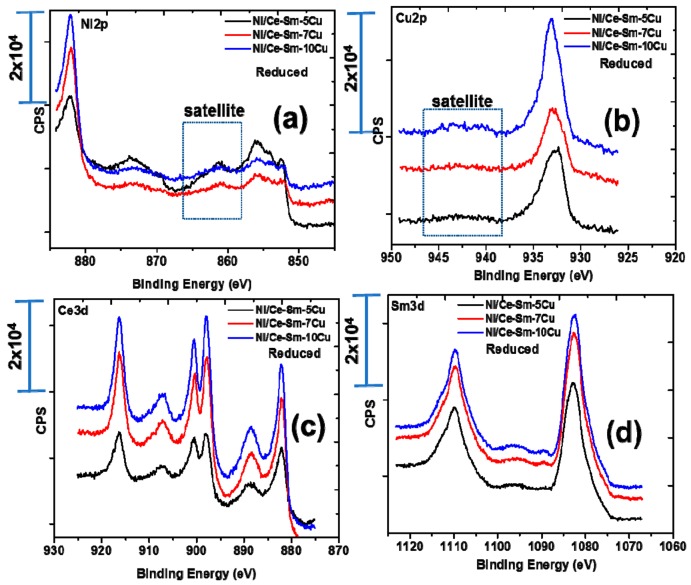
Core level spectra of: (**a**) Ni2p, (**b**) Cu2p; (**c**) Ce and (**d**) Sm3d, respectively of the reduced Ni/Ce-Sm-xCu catalysts.

Figure 5 and Figure 6 present the core level spectra of Ni2p (a), Cu2p (b), Ce (c) and Sm3d (d), respectively, following calcination at 800 °C for 4 h (Figure 5) and reduction at 800 °C for 1 h (Figure 6), respectively. For the calcined catalysts, the following remarks can be made regarding the oxidation states of the elements: The Ni2p profile is composed of a peak at ~854 eV which corresponds to Ni^2+^ species of the bulk NiO phase and a peak at ~856 eV accompanied by a satellite peak at 861.48 eV, which designates the presence of Ni^2+^ species in strong interaction with the support [60], most likely with Cu^2+^ species [61]. This finding corroborates the coexistence of at least two Ni^2+^ species on the surface of the calcined catalysts. Based on the Cu2p_3/2_ core level spectra, the peak at 933 eV escorted by a satellite peak in the 940–945 eV coincides with the presence of Cu^2+^ oxidation state on the surface of the catalysts, in agreement with the XRD and Raman results. Based on the Ce3d core level spectra, it can be stated that most of Ce is in Ce^4+^ oxidation state with minor Ce^3+^. The Sm 3d peak at 1082.5 eV corresponds to the Sm^3+^ oxidation state as anticipated. Following reduction of the catalysts, the presence of Ni^0^ was confirmed based on the XPS peak at 852 eV (Figure 5a). The binding energy of Cu species is shifted to slightly lower values of 932.78 eV compared to 933.28 eV in the case of the calcined Ni/Ce-Sm-5Cu catalyst (Table 2) and this can be linked with the change of the electronic density of the atoms. 

The drop of the BE of the Ni from 856.28 to 856.08 with the simultaneous increase of the BE of the Cu species from 933.28 to 933.78 is associated with charge transfer from Ni to Cu [51,52]. It is important to notice that the applied reduction conditions did not lead to complete reduction of the catalyst, and thus, co-existence of Ni^2+^ and Ni^0^ species was found after the reduction. Deconvolution of the reduced Ni catalysts spectra allowed quantification of Ni^0^, where it was found that the % of Ni^0^ is reduced as the Cu content is increased. Regarding the surface atomic concentrations, it was found that the catalysts surface presents a Cu- enrichment accompanied by a Ni, Ce, Sm-deficiency. The Cu enrichment takes place to a greater extent in reducing atmosphere. 

**Figure 7:**
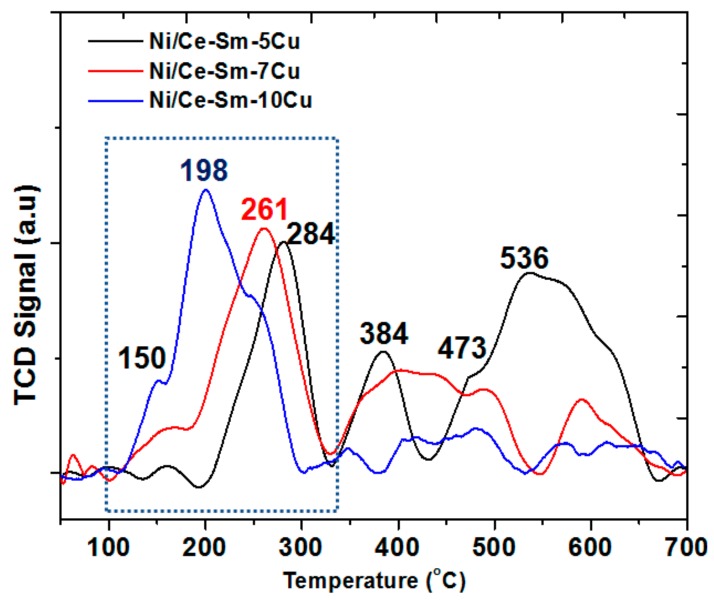
H_2_ temperature-programmed reduction (H_2_-TPR) profiles of the Ni/Ce-Sm-xCu (x = 5, 7, and 10 at.%) catalysts.

The TPR profiles of the Ni/Ce-Sm-5Cu, Ni/Ce-Sm-7Cu and Ni/Ce-Sm-10Cu catalysts are presented in Figure 7. The TPR profiles are dominated by peaks in three different regions, namely 100–300 °C, 300–500 °C and >500 °C, demonstrating the presence of different species of mobile oxygen in these catalysts in the surface and in the bulk, a property of particular importance in steam reforming reaction [62,63]. Due to the multi-elemental composition of the catalysts, and the multiple peaks apparent in the patterns, which corroborate for the reduction process complexity, it is useful to firstly present some useful considerations, based on the literature, as following:

1. According to the literature [64], for a 10Cu/CeO_2_ catalyst, two reduction peaks at 200 °C and 240 °C were found, which correspond to the reduction of highly dispersed CuO nano-particles on CeO_2_ (peak at 200 °C), and bulk CuO entities [64], respectively.
2. In the case of 10Ni/CeO_2_ catalyst, two reduction peaks were found at higher temperatures, compared to the 10Cu/CeO_2_ catalyst, namely at 271 °C and 357 °C. Also, for the 10Ni/CeO_2_ catalyst, the Ni^2+^ to metallic Ni^0^ reduction took place at 357 °C. In the case where some of the Ni^2+^ ions have been incorporated into ceria lattice, then their reduction took place at higher temperatures (425–450 °C). Herein, this broad peak was noticed only in the case of Ni-Ce-Sm-7Cu and a Ni/Ce-Sm-10Cu catalyst, which implies that in these cases, incorporation of Ni into the ceria fluorite structure, might have happened to a greater extent compared to the Ni/Ce-Sm-5Cu catalyst.
3. According to many literature reports, the reduction of single ceria takes place at ≈500 and 830 °C for the surface and the bulk oxygen species, respectively [52].
4. The low temperature reduction peak at 150 °C can be linked to the reduction of amorphous CuO, in weak interaction with the support, whereas in the 150–200 °C range, the reduction of CuO in strong interaction with the support and the partial reduction of surface CeO_2_ at the metal support interface takes place [53,65,66]. The peak above 200 °C can be assigned to highly dispersed NiO [53,65,66]. Among the catalysts presented in Figure 7, the Ni/Ce-Sm-5Cu has the highest reduction temperature (284 °C) compared to the Ni-Ce-Sm-7Cu (261 °C) and Ni-Ce-Sm-10Cu (198 °C) and this can be due to the Cu-rich character of this catalyst (Ni/Cu = 0.37) corroborating for some incorporation of the Ni into the ceria fluorite structure, and thus, suppressing its reduction. However, such an Ni incorporation is expected to diminish the sintering likelihood for this catalyst.
5. It has been reported by Lin et al. [67] that the TPR of the CuNi bimetallic catalysts had five TPR peaks, demonstrating the complexity of the reduction process. It was also suggested that the presence of metallic Cu enhanced the reduction of the Ni. This effect might be due to the competitive growth of the two oxide phases (NiO, CuO) that leads to a reduction in their crystallite size. It is worthwhile to recall here that in Figure 1 (XRD data) only traces of NiO and CuO phases were found. In agreement with Lin et al. [67], easiest reduction took place in the case of Ni/Ce-Sm-10Cu.

Given the above considerations, from the H_2_-TPR profiles presented in Figure 7, it can be argued that the peak at the lowest temperature region can be assigned to the reduction of the NiO located on the support surface, while the peaks at the medium and high temperature region can be attributed to the Ni^2+^ ions located in the metal-support interface and support reduction. It is worthwhile to mention here that as the Cu content in the support increased, the Ni reduction peak shifted to a lower temperature, as discussed above, which corroborates with a weaker metal-support interaction. Based on the low regime reduction peaks in the profiles presented in Figure 7, it can be stated that the combination of Ni and Cu in different ratios has significantly facilitated the reduction temperature of both Ni and Cu.

#### 3.1.4. Surface Acidity/Basicity Studies

The study of surface acidity/basicity is of pivotal importance as it has been reported that during the glycerol steam reforming, the gas products increase and the liquid ones decrease when the surface is more enriched with basic sites [13,14,15]. The liquid products distribution is well correlated with the distribution of the surface acid-basic sites as has been proposed by Simonetti and Dumesic [68]. It is known that in bi-functional catalysts, the presence of both acid or basic sites facilitate dehydration, whereas metal sites enhance hydrogenation reactions. For example, the production of acetone in the liquid products is expected to be increased with the basicity of the catalyst, whereas it is expected that the basic sites favor the propanediol formation from hydroxyacetone, and the hydrogenation of propanediol to acetone. Also, the acid sites promote the successive dehydrogenation of the glycerol-derived fragments, thus facilitating the coke deposition on the catalysts surface. 

**Figure 8:**
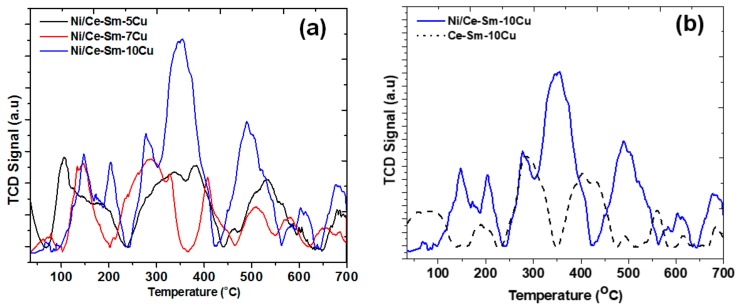
CO2-Temperature Programmed Desorption (CO2-TPD) profiles over the: (**a**) Ni/Ce-Sm-xCu (x = 5, 7 and 10 at.%) catalysts; (**b**) Ni/Ce-Sm-10Cu catalyst and Ce-Sm-10Cu support.

Figure 8a presents the CO_2_-TPD profiles of the herein studied Ni catalysts. There are three regimes of CO_2_ desorption in the 100–600 °C range. The weak, medium and strong basic sites can be tracked in the temperature ranges of 120–240 °C, 250–420 °C and >500, respectively. As can be seen, the Ni/Ce-Sm-5Cu catalyst possesses a broad low temperature peak which suggests heterogeneity of basic sites with similar affinity to CO_2_ (close activation energy of CO_2_ adsorption) and two maxima at 100 and 200 °C of CO_2_ desorption. The Ce-Sm-7Cu possesses the weakest basic sites as its bimodal basic sites distribution has maxima at 75 and 150 °C. Finally, for Ni/Ce-Sm-10Cu, the bimodal distribution of the weak basic sites has maxima at 150 and 200 °C, whereas it presents the highest population of medium and strong basic sites. For comparison purposes, the CO_2_-TPD profiles for the Ni/Ce-Sm-10Cu catalyst and the stand-alone support (Ce-Sm-10Cu) are presented in Figure 8b. It is obvious that the incorporation of Ni led to a redistribution of the basic sites and an increase of the weak (100–220 °C) and strong (>500 °C) basic sites.

**Figure 9:**
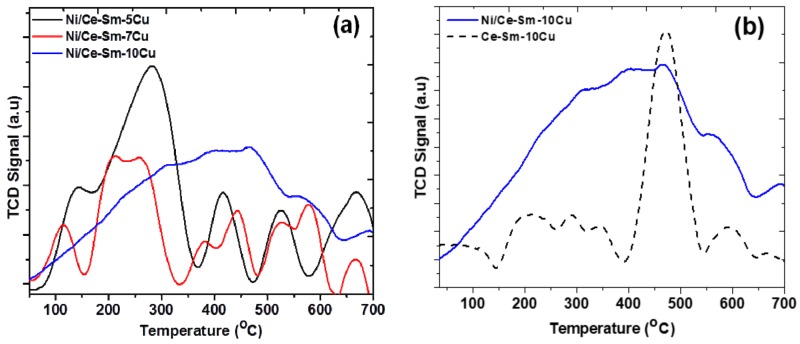
NH_3_-TPD profiles over the: (**a**) Ni/Ce-Sm-xCu (x = 5, 7 and 10 at.%) catalysts; (**b**) Ni/Ce-Sm-10Cu catalyst and Ce-Sm-10Cu support.

As can be seen in Figure 9a, for all catalysts herein, the acidic sites can be tracked in three desorption regions, namely at 100–300 °C, 300–500 °C and >500 °C, corroborating for the presence of weak, medium and strong acid sites [69]. This acidity profile is in agreement with the literature [70], where three peaks were reported by the authors in the 100–750 °C range, namely in the 120–280 °C, 280–450 °C and 450–750 °C, respectively. CeO_2_ in general has been reported to increase the acid sites on a surface. Also, regarding the Ni addition, it has been reported that Ni addition can increase the acidity compared to the bared supports in the case of CeO_2_–ZrO_2_/ SiO_2_ supports, whereas in the case of alumina and silica-alumina, both trends have been reported (increase [71] and decrease [72]). It is worthwhile to mention that in the case of Ni/Ce-Sm-10Cu the NH_3_-TPD profile is dominated by a broad multi-peak reduction profile supporting the plurality of NH_3_ adsorption sites with similar affinity for the adsorbate. In the case of Ni/Ce-Sm-5Cu, there is a bimodal distribution of weak and medium sites, whereas for the Ni/Ce-Sm-7Cu catalyst, there is a trimodal and bimodal distribution of weak and medium acid sites, respectively. The Ni/Ce-Sm-5Cu has the highest population of weak acid sites. For comparison purposes, the NH_3_-TPD profiles for the Ni/Ce-Sm-10Cu catalyst and the stand-alone support are presented in Figure 9b. It is obvious that the introduction of Ni through the wet impregnation method led to a reduction of the weak and medium acid sites predominantly.

### 3.2. Catalytic Performance

#### 3.2.1. Glycerol Conversion and Selectivity to Gaseous Products

**Figure 10:**
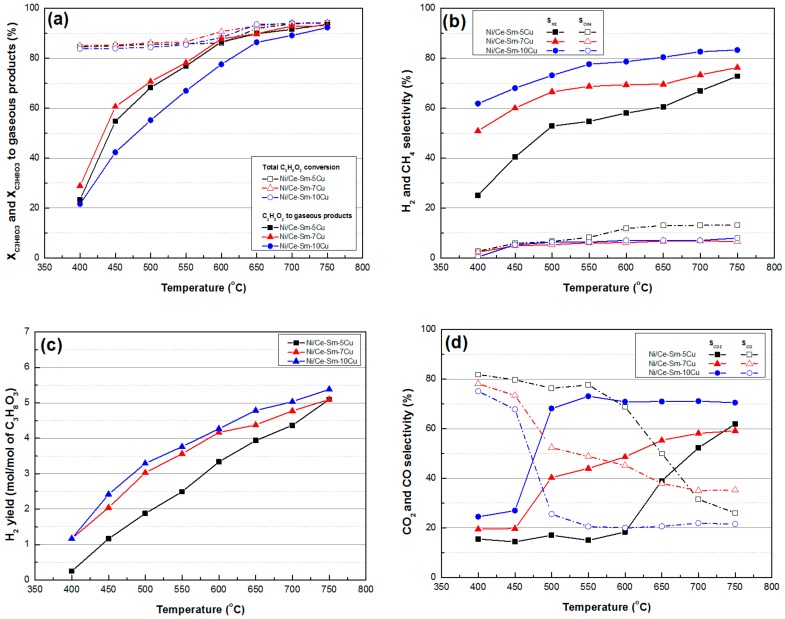
(**a**) Total glycerol conversion and glycerol conversion into gaseous products; (**b**) H_2_ selectivity and CH_4_ selectivity; (**c**) H_2_ yield; (**d**) CO_2_ and CO selectivity [Results obtained for samples tested under experimental protocol #1].

The effect that the supporting material had on the catalytic activity and selectivity of the Ni/Ce-Sm-xCu catalysts was initially investigated in the temperature range 400–750 °C, using a WGFR = 20:1, molar (experimental protocol #1). The reproducibility of the experimental results was tested by repeating all experiments at least three times and calculating 95% confidence intervals for the mean value. It was found that individual experimental values lay well within the corresponding confidence intervals showing very good reproducibility of the repeated experiments (Figure S1). In terms of total glycerol conversion (X_C3H8O3_), it is clear that all catalysts had a similar performance, showing very high X_C3H8O3_ for the entire temperature range under investigation; these values ranged from ≈84% at 400 °C to ≈94% at 750 °C (Figure 10a). Due to the endothermic nature of the reaction, glycerol conversion to gaseous products (X_C3H8O3-gas_, %) strongly increases with temperature, however, although the Ni/Ce-Sm-5Cu and Ni/Ce-Sm-7Cu exhibit only marginal differences between them, the further increase in Cu content appears detrimental to performance, especially between 450–650 °C, as evidenced by the lower X _C3H8O3_-gas observed for the Ni/Ce-Sm-10Cu catalyst (Figure 10a). In any case, all bimetallic samples have proven to be more active than the Ce-Sm-10Cu, which reveals much lower values for the X_gaseous_ for the whole temperature range (a comparison between Ni/Ce-Sm-10Cu and Ce-Sm-10Cu will be shown in Figure 12, below). A similar observation was reported by Liu et al. [61] for the selective hydrogenolysis of biomass-derived xylitol to ethylene glycol and propylene glycol reaction. As a matter of fact, the NiCu-SiO_2_ bimetallic catalysts showed much superior activities and selectivities to the target glycols relative to the monometallic Cu-SiO_2_ and Ni-SiO_2_ catalysts; enhanced performance was ascribed to their significantly promoted C–OH dehydrogenation and C=O hydrogenation activities and their high resistance to sintering imposed by the structural and electronic effects of Ni.

The influence of reaction temperature on hydrogen and methane selectivity (S_H2_ and S_CH4_, respectively) is shown in Figure 10b. With regards to S_H2_, it follows the trend Ni/Ce-Sm-10Cu > Ni/Ce-Sm-7Cu > Ni/Ce-Sm-5Cu, i.e., the higher the Cu content, the more selective towards H_2_ the catalysts appears to be. The same is also true for hydrogen yield, Y_H2_ (Figure 10c) with values equal to 5.4 (Ni/Ce-Sm-10Cu), 5.1 (Ni/Ce-Sm-7Cu) and 5.0 (Ce-Sm-5Cu), at 750 °C. In terms of S_CH4_, the Ni/Ce-Sm-7Cu and Ni/Ce-Sm-10Cu samples exhibit low, stable values for the entire temperature range (Figure 10b). In contrast, for the Ni/Ce-Sm-5Cu catalyst, these values increase with temperature from ≈3% at 400 °C to 13% at 750 °C

It has been reported that by using high WGFR (as in our case), the formation of CH_4_ is inhibited at reaction temperatures higher than 650 °C due to the influence of the methane steam reforming reaction (Equation (10)) [73]. It has also been reported in the literature [74,75] that the use of Cu in the reforming processes can suppress the methanation reaction (Equation (11)). Moreover, Kawi et al. [64] proposed that the formation of Ni-Cu alloy can prevent CO dissociation by enhancing CO adsorption to suppress methanation. Additionally, Lin et al. [67] argued that intermediates such as methoxy and formyl species can be reformed efficiently over Cu surfaces to produce H_2_ and consequently reduce CH_4_ reformation. As for the CeO_2_-Sm_2_O_3_ supporting material, it has been reported that CO_2_ can react with Sm_2_O_3_ to form Sm_2_O_2_CO_3_, which then reacts with carbon to produce CO [64].

CH_4_(g) + H_2_O(g) ↔ CO(g) + 3H_2_(g), (10)

CO_2_(g) + 4H_2_(g) ↔ CH_4_(g) + 2H_2_O(g), (11)

CO_2_ and CO selectivity values are depicted in Figure 10d. At low reaction temperatures, all catalysts are more selective towards CO and less selective towards CO_2_; however, this behaviour changes as temperature increases. Specifically, for the Ni/Ce-Sm-10Cu catalysts, S_CO_ shows a sharp drop after 450 °C and then stabilises at around 20% up to 750 °C. Consequently, S_CO2_ increases greatly after 450 °C and remains stable at ≈70% up to 750 °C. The same trend can also be observed for the Ni/Ce-Sm-7Cu catalyst, but the decline in S_CO_ values (and increase in S_CO2_) is less sharp and continuous up to 700 °C, where it appears to be stabilised. The decline in the S_CO_ (increase in S_CO2_) for the Ni/Ce-Sm-5Cu catalyst was shifted by approximately 100 °C (above 550 °C) and remained rather precipitous up to 750 °C. The tendency to produce CO at lower temperatures may be attributed to the prevalence of the reverse water gas shift reaction (Equation (2)) while, as the temperature increases, the endothermic Boudouard reaction (Equation (12)) seems to predominate.

2CO(g) ↔ CO_2_(g) + C(s), (12)

These findings are in accordance with Khzouz et al. [76], suggesting that the bimetallic Ni-Cu catalyst had a strong influence on the amount of CO_2_ and CO produced, due to the different selectivity towards the WGS and decomposition reactions, revealing that Cu alloying in Ni catalyst had an inhibiting effect for CO and/or CO_2_ hydrogenation. Moreover, according to Saw et al. [64], a Ni–Cu/CeO_2_ catalyst with Ni/Cu ratio of 1 exhibited a high reaction rate with the least methane formation due to the formation of the Ni–Cu alloy phase, as it was found to be the active site for WGS reaction with methane suppression. 

Thus, it can be concluded that the presence of Cu in the catalysts resulted in the suppression of the undesirable methanation side-reaction, while the Ni component was important for the high WGS activity. These findings strongly suggest that the bimetallic Cu–Ni compositions are highly promising as GSR catalysts.

#### 3.2.2. Selectivity to Liquid Products

**Figure 11:**
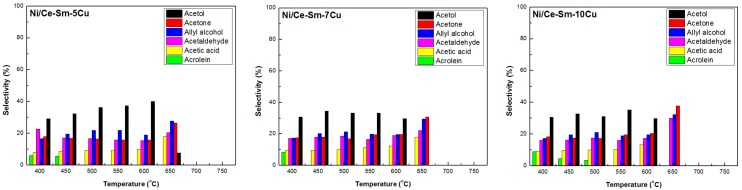
Liquid products selectivity for the Ni/Ce-Sm-xCu (x = 5, 7, and 10 at.%) catalysts [Results obtained for samples tested under experimental protocol #1].

Although trace amounts of a variety of liquid products were detected, only the main ones, i.e., acetol, acetone, allyl alcohol, acetaldehyde, acetic acid, and acrolein were quantified. In general, all catalysts stopped producing effluents over 650 °C and the main product was acetol up to 600 °C (Figure 11). Acrolein was produced only at low temperatures, i.e., 400–450 °C for the Ni/Ce-Sm-5Cu, 400 °C for the Ni/Ce-Sm-7Cu and 400–500 °C for the Ni/Ce-Sm-10Cu. Moreover, for all samples, the production of allyl alcohol and acetaldehyde was quite stable for all temperatures, showing a slight increase at 650 °C. Acetic acid was produced up to 650 °C for the Ni/Ce-Sm-5Cu and Ni/Ce-Sm-7Cu and up to 600 °C for Ni/Ce-Sm-10Cu. So the main difference between the samples is the non-production of acetol and acetic acid at 650 °C for the Ni/Ce-Sm-10Cu catalyst. As for the Ce-Sm-10Cu sample, a variety of liquid effluents were produced even at high reaction temperatures. Specifically, for T equals to 750 °C, allyl alcohol (35%), acetaldehyde (30%), acetic acid (19%) and acetone (16%) were the liquid products determined, whereas acrolein was produced for temperatures as high as 500 °C.

According to the literature [33,35,45,46], glycerol can dehydrate through two distinct and independent pathways, one leading to acetol and the other forming acrolein, through 3-hydroxypropenal (3-HPA), a very unstable product. The first pathway implies the removal of one of the two terminal alcohol groups in the glycerol molecule, whereas the second implies the removal of the central alcohol function [77,78]. Moreover, the proposed reaction mechanism for the glycerol hydrogenolysis involves its dehydration to acetol, which is assumed to occur preferentially in acid sites [79,80,81,82], and simultaneously reformed to H_2_ and CO_2_, in metal sites. This hydrogen generated in situ could be used for the hydrogenation of acetol to form propylene glycol in metal sites. Furthermore, ethanol can be produced from the propylene glycol hydrogenolysis [80,83] whereas acrolein may form by excessive dehydration of glycerol [84,85].

**Figure 12:**
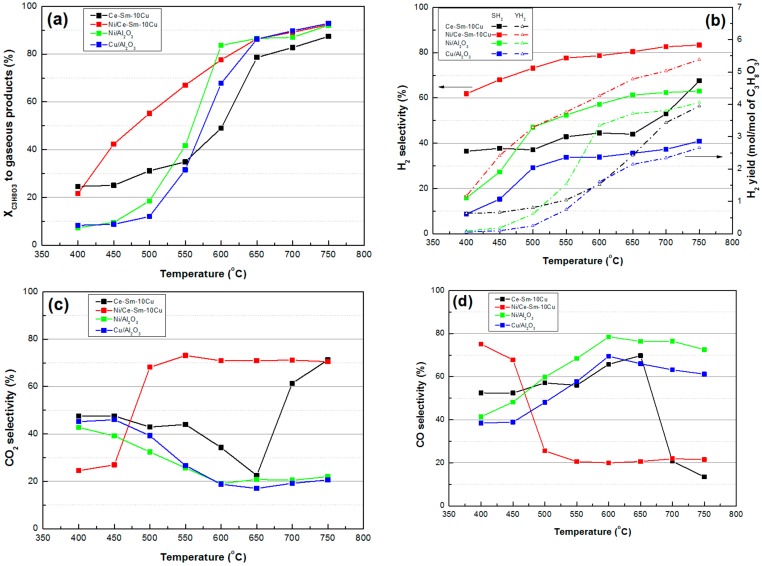

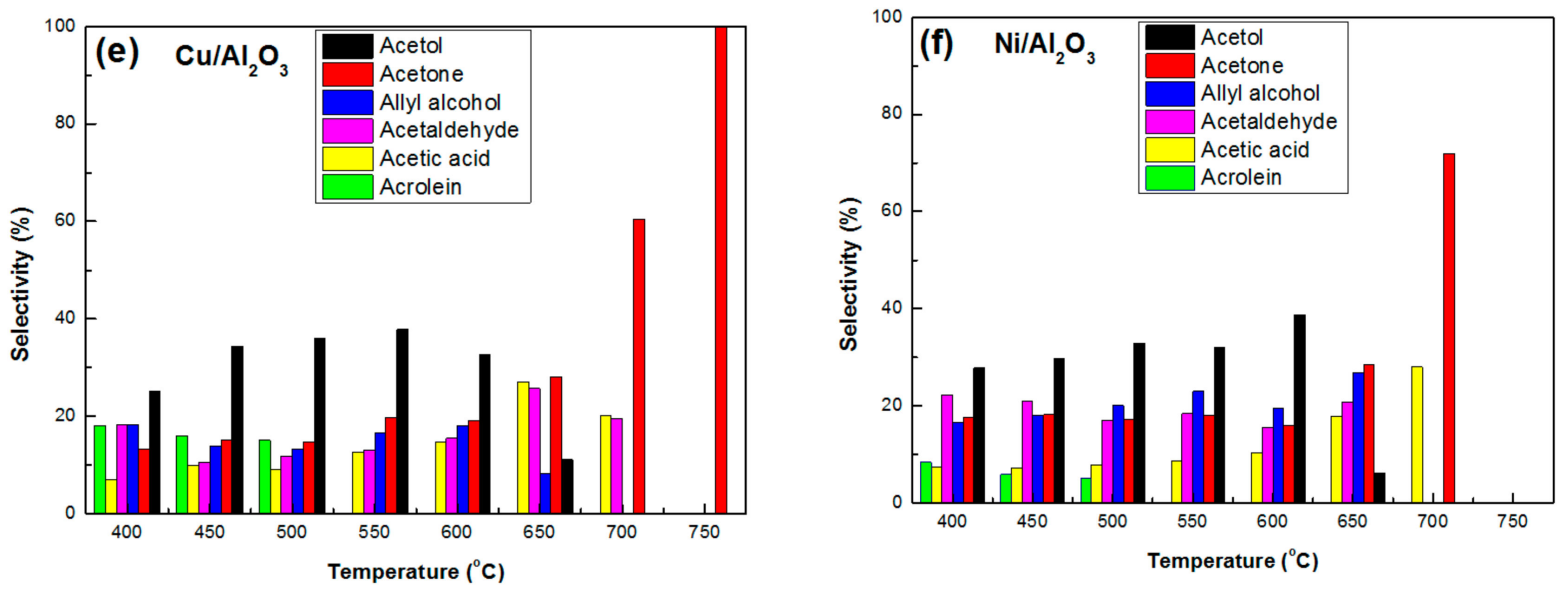
Comparative results obtained over the Ni/Ce-Sm-10Cu catalyst, the stand alone Ce-Sm-10Cu support and representative monometallic catalysts (Ni/Al_2_O_3_ and Cu/Al_2_O_3_): (**a**) Conversion to gaseous products; (**b**) H_2_ selectivity; (**c**) CO_2_ selectivity; (**d**) CO selectivity; (**e**) Selectivity of liquid products over Cu/Al_2_O_3_; and (**f**) Selectivity of liquid products over Ni/Al_2_O_3_ [Results obtained for samples tested under experimental protocol #1].

In Figure 12, a comparison of the Ni-Ce-Sm-10Cu catalytic performance with the Cu-Sm-10Cu and other monometallic samples (Ni/Al_2_O_3_, Cu/Al_2_O_3_), in terms of key reaction metrics for various temperatures, can be seen. Please note that results for the Ni/Al_2_O_3_, Cu/Al_2_O_3_ have been previously reported in Reference [30]. It is observed that the bimetallic sample exhibits the highest activity for glycerol conversion to gaseous products amongst all samples, especially for the low T range. It also exhibits the highest values for H_2_ yield (~5.5 at 750 °C), S_H2_ (60–85%) and S_CO2_ (70% for 500 °C >T>750 °C), whereas S_co_ has the lowest value (20%) for the same temperature range. As for the liquid products selectivity, it can be seen that for both Ni/Al_2_O_3_, Cu/Al_2_O_3_ samples, acetone was the main one for temperature, being as high as 700 to 750 °C, whereas for all bimetallic catalysts, no liquid was produced for the same T range.

Thus, it can be concluded that the decoration of Cu nanoparticles by a second metal like Ni with high H_2_ activation capability can be an effective strategy to combine the high selectivity of the Cu metal and the high hydrogenation capacity of the metal promoter, which would result in an efficient bimetallic catalyst for the GSR reaction.

### 3.3. Catalytic Stability

**Figure 13:**
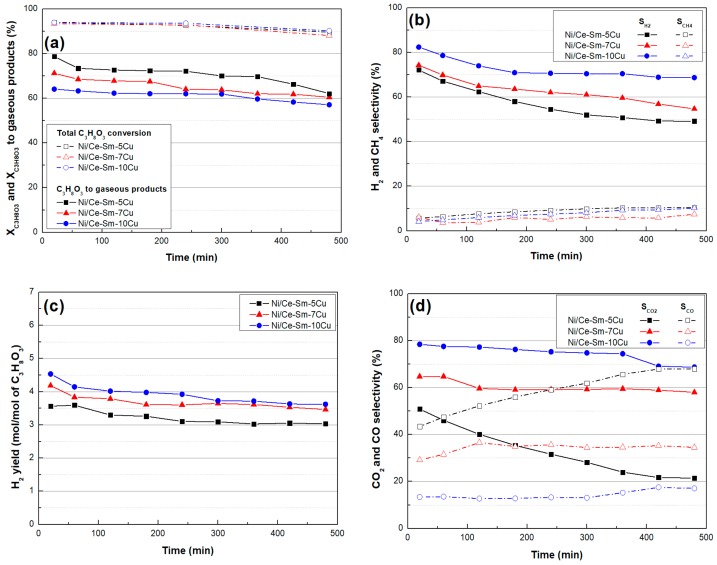
Time on stream experiments for all catalysts: (**a**) Total glycerol conversion and glycerol conversion into gaseous products; (**b**) CH_4_ selectivity; (**c**) H_2_ yield; (**d**) CO_2_; and CO selectivity [Results obtained for samples tested under experimental protocol #2].

**Figure 14:**
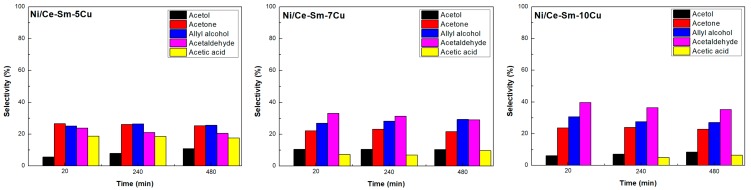
Liquid products selectivity for the Ni/Ce-Sm-xCu (x = 5, 7, and 10 at.%) catalysts [Results obtained for samples tested under experimental protocol #2].

The time on stream experimental results are presented in Figure 13 and Figure 14. From Figure 13a, it can be depicted that all catalysts deactivate at a rather slow rate, approaching the same values for glycerol conversion (~90%) and for conversion into gaseous products (~60%) after 8 h of time on stream. The variation of H_2_ and CH_4_ selectivity with reaction time can be seen in Figure 13b. It can be depicted that S_H2_ gradually decreases with time for all samples with the Ni/CeSm10Cu exhibiting the highest value (~70%) between them, whereas S_CH4_ remains at low level values during the whole reaction time (<10%). 

Moreover, the H_2_ yield (Figure 13c) reaches the value of 3.5 and 3.0 mol H_2_/mol glycerol for the Ni/Ce-Sm-10Cu, Ni/Ce-Sm-7Cu and the Ni/Ce-Sm-5Cu sample, respectively. From Figure 13d, it can be seen that the catalysts with the higher Cu loading were more selective for CO_2_ and less selective for CO for the whole period of time; the Ni/Ce-Sm-10Cu sample reveals the highest S_CO2_ (70–80%) and lowest S_CO_ (10–20%) among them. As for the Ni/Ce-Sm-5Cu sample, an almost equimolar mixture was produced at the beginning of the experiment, whereas S_CO2_ was decreased (from 50% to 20%) and S_CO_ was increased (from 45% to 70%) with time on stream. As for the liquid products, it can be seen (Figure 14) that their selectivity values were quite constant with time for all catalysts; acetaldehyde and acetic acid were the ones with the highest (35–40%) and lowest values (5–10%) for the Ni/Ce-Sm-10Cu and Ni/Ce-Sm-7Cu samples. In contrast, acetone and allyl alcohol (both at 30%) were the main liquid products for the Ni/Ce-Sm-7Cu sample, with acetol being the minor one (5–10%). The above findings can be explained on the basis of possible transition metal alloy formations that are generally used in heterogeneous catalysis reactions due to their unique properties, as compared to pure transition metals [74]. In fact, the electronic and geometric structures of alloy structures have proved to have great effects toward catalyst reactivity and selectivity [86,87]. Specifically, Ni–Cu alloys are currently receiving great interest in many chemical reactions such as steam reforming of methane/ethanol/dimethyl ether, methane decomposition, and hydrogenation reactions using CO_2_, CO, dimethyl oxalate and others [88,89,90,91,92]. De Rogatis et al. [93] reported that Ni–Cu alloy catalyst is able to improve catalyst stability in steam reforming of ethanol reaction. Chen and Lin [94] also showed that interaction between Ni–Cu is able to achieve high performance in ethanol and acetaldehyde conversion, as well as high selectivity and stability.

Thus, it can be suggested that in the glycerol steam reforming (GSR) process, the catalysts contribute, exhibiting two different effects [95]. The first is to promote the cleavage of the C–H, C–C and O–H bonds in the reactant molecule to form some small fragments, and then the fragments should be recombined to produce gaseous products, such as CO, CO_2_, CH_4_ and H_2_. The second one is to change reaction paths of the side-reactions, such as methanation reaction, methane steam reforming and water gas shift (WGS) reaction. It has also been reported that Ni-based catalysts are susceptible to deactivation because the active nickel species are easily sintered and the coke is easily formed on the catalyst surface to cover the active sites of the catalyst during the process of GSR [14,15,16]. The sintering of nickel particles can rapidly decrease catalytic activity, but it can be suppressed by adding a second metal to form different alloys. Therefore, Ni-based bimetallic catalysts, such as Ni-Cu seem worthwhile to be investigated in order to evaluate the effect of the second metal on the anti-sintering and the anti-carbon deposition. 

### 3.4. Characterization of Used Catalysts

**Figure 15:**
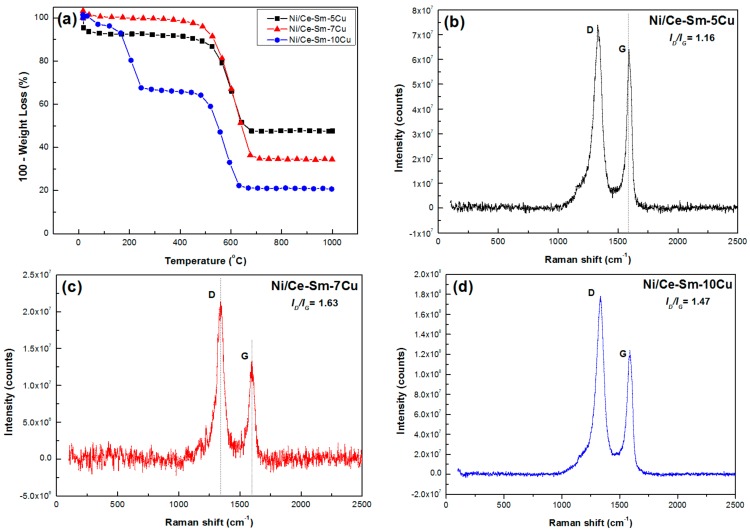
(**a**) TGA profiles of the Ni/Ce-Sm-xCu (x = 5, 7, and 10 at.%) catalysts; Raman spectra of the used: (**b**) Ni/Ce-Sm-5Cu (**c**) Ni/Ce-Sm-7Cu, and (**d**) Ni/Ce-Sm-10Cu catalysts.

The carbon deposited on the spent catalysts that were tested under experimental protocol #2 were examined using TGA and Raman spectroscopy (Figure 15). From the results of TGA analysis (Figure 15a), it is clear that high amounts of carbon were deposited on all samples following the order Ni/Ce-Sm-5Cu < Ni/Ce-Sm-7Cu < Ni/Ce-Sm-10Cu (52, 65 and 79 wt.%, respectively). For the Ni/Ce-Sm-5Cu and Ni/Ce-Sm-7Cu spent catalytic samples, the oxidation process takes place between ≈450–700 °C (main peak at ≈600 °C). As accepted in the literature, oxidation peaks below 500 °C can be attributed to easily combustible amorphous carbon species, while those between 500–700 °C correspond to the oxidation of graphitic carbon allotropes [96,97]. For the Ni/Ce-Sm-10Cu, a second low temperature event centred around 200 °C can also be observed, which can be ascribed to the desorption of hydrocarbon species absorbed on the catalysts surface [98]. In addition, the main thermal event seems to have shifted at lower temperatures (by about 50 °C). Thus, although large amounts of carbon were deposited on all catalysts, the majority combust at temperatures below 600 °C, which helps explain the excellent catalytic performance of all samples. 

It can be speculated that glycerol dehydration in the acid sites is the reaction rate controlling step, as the acetol formed readily interacts with the hydrogen species formed from molecular hydrogen, being rapidly hydrogenated in the metal sites. Hence, although the number of active metal sites is reduced with time on stream due to coke formation, there are still enough remaining ones for the fast hydrogenation of the acetol [99].

The results from Raman spectroscopy are depicted in Figure 15b–d. The spectra show two broad bands, the D-band centred around 1350 cm^−1^ and the G-band detected around 1580 cm^−1^. The D-band is the result of a disorder-induced double resonant process, which is caused by the breakdown of the usual wave vector selection rule (A_1g_ symmetry), and is associated with the disordered structural mode of crystalline carbon species. The G-band, located at the centre of the Brillouin zone (BZ), corresponds to the in-plane optical mode of vibration (E_2g_ symmetry) of two neighbouring carbon atoms on the perfect hexagonal graphite and is associated with graphitic carbon with high degree of symmetry [100,101]. The relative intensity of these two bands (*I_D_/I_G_*) can provide valuable information regarding the degree of crystallinity of the carbon formed during the GSR, as smaller *I_D_/I_G_* values indicate higher crystallinity due to higher contribution of the graphitized carbon formed [102,103]. For the catalysts tested herein, it followed the order 1.16 < 1.47 < 1.63 for the Ni/Ce-Sm-5Cu, Ni/Ce-Sm-10Cu and Ni/Ce-Sm-7Cu, respectively. Thus, the Raman results suggest that the incorporation of Cu in the support matrix helps control the graphitisation degree of the carbon deposited during GSR at the given conditions studied (8 h, 650 °C). This can be interpreted as an alteration of the structure of Ni sites that are responsible for the nucleation and growth of the graphitic structures. Though the above trend should be considered with caution, as it can be changed with increasing the time of stream and temperature due to sintering phenomenon and enhanced de-hydrogenation that will affect the kinetics of the graphitization process. 

The above findings are in accordance with Zhang et al. [104], who reported that bimetallic catalysts Cu–Ni, Co–Ni, and Pt–Ni did not show any C_2_ or C_3_ species on their surface during the steam reforming of ethanol. Additionally, lower reaction temperature and a higher amount of amorphous carbon have been reported, compared to that for supported nickel nanoparticle catalysts; the carbon deposited is considered to be formed by decomposition of methane. The decomposition of methane, most apparently occurs if the high density of nickel d-state, which is near to Fermi level, interacts with the vacant anti bonding orbitals of C–H bond [105].

Moreover, Fierro et al. [106] suggested that with the addition of Cu, the large ensembles of Ni metal atoms are extinguished, melting temperature turns out to be average, the existing state of Ni is altered, and the affinity for carbon changes. As a result, a NiCu solution is formed, which results in lower deposition of carbon by CH_4_ decomposition [107,108].

Thus, it can be stated that although Cu metal is effective for C–O hydro-dehydrogenation as mentioned above, it is not very active for H_2_ activation, most likely due to its lower binding energy to hydrogen (−2.39 eV) in comparison with other transition metals, such as Ni and Co with much stronger binding (−2.89 eV) [109,110]. As a result, Cu catalysts generally present inferior activities for C=O and C=C bond hydrogenation in comparison with Ni catalysts [111,112]. In the context of this work, it has also been proven that CeO_2_ promoted with Sm_2_O_3_ can be used as supporting material for the GSR reaction producing H_2_ with very low CO concentration, as they are capable to tune redox properties of the catalysts, which may alleviate coking from CO disproportionation. In addition, it has been suggested that transition metals (as Cu) can be employed to manipulate the catalytic activity of Ni through alloying or forming bi-metallic sites.

## 4. Conclusions

In the present study, Ni/Ce-Sm-xCu (x = 5, 7, 10 at.%) catalysts were synthesized using microwave radiation coupled with sol-gel and followed by the wetness impregnation method for the Ni metal incorporation. The catalysts were evaluated for the glycerol steam reforming and the following remarks can be drawn:

- The catalysts are mainly composed of ceria type cubic lattice with traces of CuO and NiO being rather non-porous or macroporous materials with a spongy morphology due to the evaporation of gases originating from the decomposition of organic compounds used in the synthesis. They also present a rich population of mobile oxygen species both in surface and in the bulk. The increase in Cu content seems to facilitate the reducibility of the catalyst. Furthermore, all catalysts present weak, medium and strong acid and basic sites, a key feature towards the tailoring of the liquid products of this reaction.
- In terms of catalytic activity, all of the catalysts had very high X_C3H8O3_ for the entire temperature range; from ≈84% at 400 °C to ≈94% at 750 °C. Ni/Ce-Sm-10Cu catalyst showed lower X _C3H8O3-gas_ implying the increased Cu content had a detrimental effect on performance, especially below 650 °C. In terms of S_H2_ and Y_H2_, both appeared to vary in the following order Ni/Ce-Sm-10Cu > Ni/Ce-Sm-7Cu > Ni/Ce-Sm-5Cu, where the high impact of Cu content is demonstrated. Moreover, the catalysts with the higher Cu content (Ni/Ce-Sm-7Cu and Ni/Ce-Sm-10Cu) had low, stable values of S_CH4_, for the entire temperature range. In contrast, for the Ni/Ce-Sm-5Cu catalyst, these values increased with temperature from ≈3% at 400 °C to 13% at 750 °C. At low reaction temperatures, all catalysts were more selective towards CO and less selective towards CO_2_. A variety of liquid products were detected, however, all catalysts stopped producing effluents over 650 °C.
- The stability testing experiments showed that the catalysts were quite stable, exhibiting high glycerol conversion (~90%) after 8 h of operation, whereas S_H2_ gradually decreased with time for all samples, with Ni/Ce-Sm-10Cu exhibiting the highest value (~70%) among them.
- All catalysts accumulated high amounts of carbon, following the order Ni/Ce-Sm-5Cu < Ni/Ce-Sm-7Cu < Ni/Ce-Sm-10Cu (52, 65 and 79 wt.%, respectively); however, the majority combusted at temperatures below 600 °C, which corroborates with the excellent catalytic performance of all samples. Raman studies over the used catalysts indicate that the incorporation of Cu in the support matrix helped control the graphitisation degree of the carbon deposited during the reaction at hand.

In conclusion, the presence of Cu in the catalysts resulted in the suppression of the undesirable methanation side-reaction, while the Ni component was important for the high WGS activity. The findings presented herein strongly suggest that bimetallic Cu–Ni compositions are highly promising as GSR catalysts.

## Figures and Tables

**Table 1:** Textural properties of the Ni catalysts studied in this work.

Catalyst	Brunauer-Emmet-Teller(BET)	D	Pore Size ^1^	Lattice Parameter ^2^	X-ray Spectroscopy (EDS) at.% (Ratios)			
	**(m^2^/g)**	**(nm)**	**(nm)**	**(Å)**	**Ce/Sm**	**Ni/Ce**	**Ni/Cu**	**Ni:Ce:Sm:Cu**
Ni/Ce-Sm-5Cu	0.68	63.9	40	5.52	1.10	0.56	5.6	0.56(22%):1(39%):0.90(35%):0.10(4%)
Ni/Ce-Sm-7Cu	0.1	62.1	198	5.51	1.04	0.86	3.23	0.86(29%):1(33%):0.96(32%):0.19(6%)
Ni/Ce-Sm-10Cu	0.7	62.3	178	5.52	1.07	0.84	2.63	0.84(27%):1(33%):0.92(30%):0.32(10%)
Ce-Sm-10Cu (support)	3.69	9.3	51.8	5.42		n/a	n/a	

^1^ Calculated by the BET method (4V/A); ^2^ using the interplanar distance of the cubic cell, d_hkl._

**Table 2:** Binding energies (eV) of the elements and surface atomic ratios based on the x-ray photoelectron spectroscopy (XPS) studies.

Catalyst	Ni2p_3/2_		Cu2p	Ce3d_5/2_	Ni/Cu	Ni/Ce	Ce/Sm
	Ni^0^	Ni^2+^			Ratio		
Ni/Ce-Sm-5Cu-c ^1^	n/a	854.08/856.08	933.28	881.78	2.81	2.66	1.15
Ni/Ce-Sm-7Cu-c ^1^	n/a	854.18/856.08	933.38	882.08	1.29	0.58	0.35
Ni/Ce-Sm-10Cu-c ^1^	n/a	854.18/855.88	933.28	881.88	0.58	0.40	0.24
Ni/Ce-Sm-5Cu-r ^2^	852.68	856.28	932.78	882.18	2.40	3.82	1.53
Ni/Ce-Sm-7Cu-r ^2^	852.48	856.08	933.08	882.18	1.50	1.70	0.71
Ni/Ce-Sm-10Cu-r ^2^	852.38	855.98	933.08	882.18	1.32	1.69	0.83

^1^ Calcined catalysts, ^2^ Reduced catalysts.

## Supplementary Materials

The following are available online at http://www.mdpi.com/2079-4991/8/11/931/s1, Figure S1: Reproducibility of the Ni/CeSm5Cu experimental results: (a) Glycerol conversion into gaseous products, (b) H_2_ yield (c) H_2_ selectivity, (d) CO_2_ selectivity, (e) CO selectivity, (f) CH4 selectivity.
