# Supplementary material for: The Effect of Ni Addition onto a Cu-Based Ternary Support on the H2 Production over Glycerol Steam Reforming Reaction
Source: Nanomaterials (Basel). 2018 Nov 8;8(11):931. doi: 10.3390/nano8110931 (PMC6266861; doi:10.3390/nano8110931)
Supplement: Supplementary file 1 [file nanomaterials-08-00931-s001.pdf]

Supporting Information

The Effect of Ni Addition onto a Cu-Based Ternary Support on the H_2 Production over Glycerol Steam Reforming Reaction

Kyriaki Polychronopoulou [1,2,*], Nikolaos Charisiou [3], Kyriakos Papageridis [3], Victor Sebastian [4,5], Steven Hinder [6], Aasif Dabbawala [7], Ayesha AlKhoori [1], Mark Baker [6], and Maria Goula [3,*]

[1] Department of Mechanical Engineering, Khalifa University of Science and Technology, Main campus, Abu Dhabi, P.O. Box 127788, UAE; ayesha.alkhoori@ku.ac.ae
[2] Center for Catalysis and Separation, Khalifa University of Science and Technology, Abu Dhabi, P.O. Box 127788, UAE
[3] Laboratory of Alternative Fuels and Environmental Catalysis (LAFEC), Department of Environmental and Pollution Control Engineering, Western Macedonia University of Applied Sciences, GR-50100, Kozani, Greece; ncharis@teiwm.gr (N.D.C.); kpapageridis@gmail.com (K.N.P.)
[4] Chemical and Environmental Engineering Department & Nanoscience Institute of Aragon (INA), University of Zaragoza, SP-50018, Zaragoza, Spain; victorse@unizar.es
[5] Networking Research Center on Bioengineering, Biomaterials and Nanomedicine, CIBER-BBN, SP-28029, Madrid, Spain
[6] The Surface Analysis Laboratory, Faculty of Engineering and Physical Sciences, University of Surrey, Guildford, GU2 4DL, UK; s.hinder@surrey.ac.uk (S.J.H.); m.baker@surrey.ac.uk (M.A.B.)
[7] Department of Chemical Engineering, Khalifa University of Science and Technology, SAN campus, P.O. Box 127788, UAE; asifdabbawala123@gmail.com

* Correspondence: kyriaki.polychrono@ku.ac.ae (K.P.); mgoula@teiwm.gr (M.A.G.); Tel. +971-(0)2-4018211 (K.P.); +30-246-106-8296 (M.A.G.)

Reproducibility of the experimental results

The reproducibility of the experimental results was tested by repeating all experiments at least three times. From these experiments, 95% confidence intervals for the mean value were calculated and the results for the Ni/CeSm5Cu catalyst in the form of error bars are depicted below. Individual experimental values lay well within the corresponding confidence intervals showing a very good reproducibility of the experimental results.

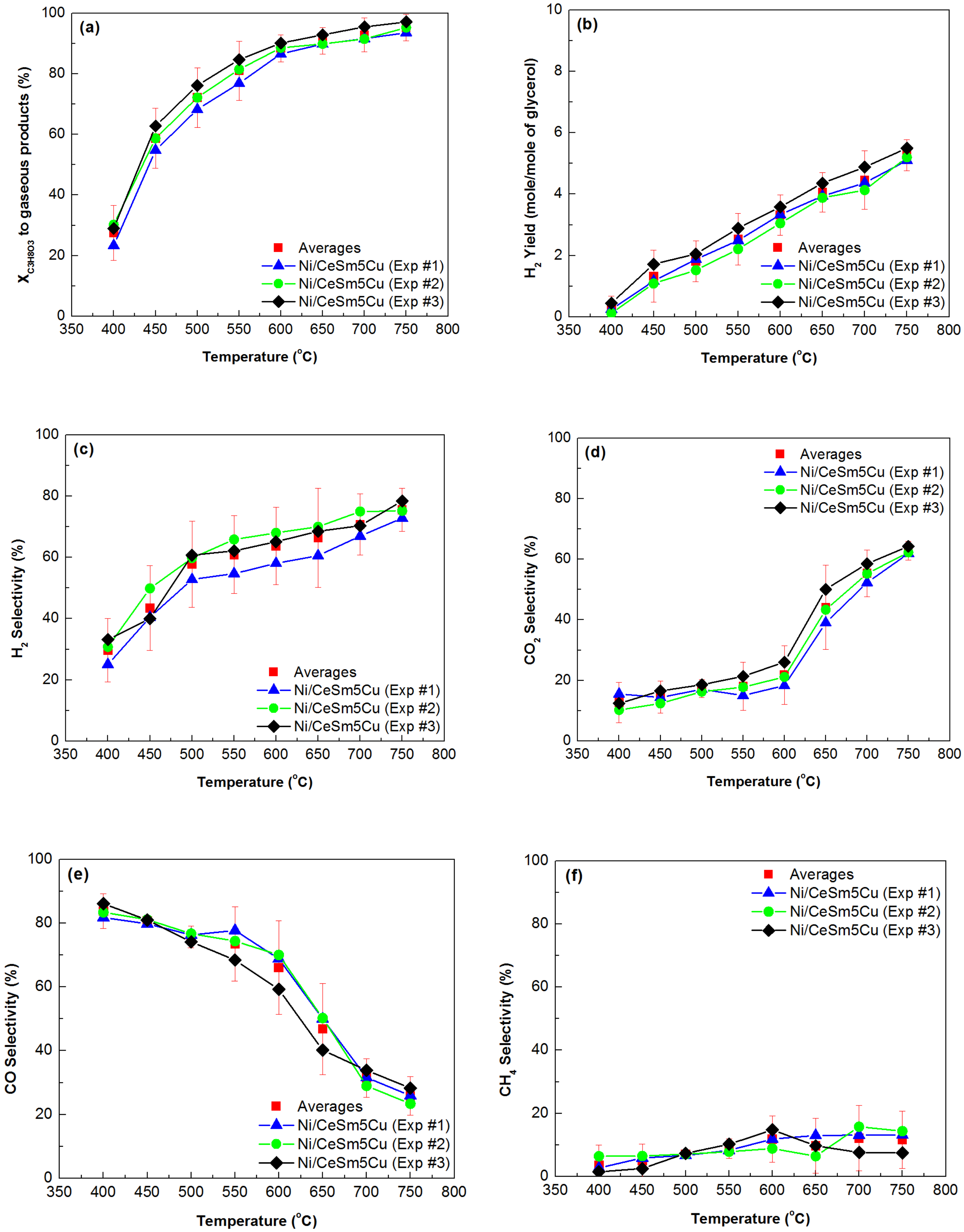

Figure S1. Reproducibility of the Ni/CeSm5Cu experimental results: (**a**) Glycerol conversion into gaseous products; (**b**) H_2 yield (**c**) H_2 selectivity; (**d**) CO_2 selectivity; (**e**) CO selectivity; **(f)** CH_4 selectivity.

Catalytic tests

The GC was calibrated for H_2, CH_4, CO_2 and CO using high purity gases (at least over 4.5) with appropriate concentrations (typically 4 to 5 concentrations were used for the calibration of each gas). The carrier gas that was used in the GC was He (5.5). In regards to the use of He as carrier gas, as is well known, hydrogen is the only element with thermal conductivity greater than helium, and mixtures of small amounts of hydrogen (<20%) in helium at moderate temperatures exhibit thermal conductivities less than either component alone. When analyzing for hydrogen with helium carrier gas, a hydrogen peak may appear as positive, negative, or as a split peak. Of course one solution to this problem is the use of nitrogen or argon-methane as carrier gas. This eliminates problems inherent with using helium as carrier, but causes reduced sensitivity to components other than hydrogen. However this issue can also be solved if the detector is operated at higher temperatures—from 200 °C to 300 °C (the detector temperature that we use is 220 °C).

In regards to the analysis procedure followed for the liquid products, the reagents and standards used where: Glycerol (≥99%), acetol (90%), acetone (≥99.8%), acetaldehyde (≥99.5%), allyl alcohol (≥99%), acrolein (≥99%), acetic acid (99.0%) and 2-butanol (≥99%). These were obtained from Sigma-Aldrich (St. Louis, MO, USA) and were used to prepare standard solutions in order to create calibration curves for each compound (typically 4 to 5 standard solutions were prepared for the calibration of each compound). The determination of liquid products was carried out on a 7890A/5975C Triple –Axis Detector diffusion pump – based GC-MS equipped with split/splitless inlet (Agilent Technologies, Santa Clara, USA). Chromatographic separation was achieved by a 30 m × 250 μm HP-5MS (5% phenyl, 95% methylpolysiloxane) capillary column with film thickness of 0.25 μm. Helium 5.0 (99.999%) was used as carrier gas at 1 mL min^{-1} in a constant flow rate mode. The oven temperature program was 35 °C for 5 min, increased by 10 °C min^{-1} to 250 °C and held at 250 °C for 10 min. The temperature of the split/splitless injector was 280 °C and the volume of the samples injected were 1 μL with a split ratio of 100:1 using ultra inert liner with glass wool (Agilent Technologies). The temperature of the ion source, the quadrupole and the MS interface for both instruments were 230, 150 and 250 °C, respectively. Both full scan (40 – 160 m/z) and Selective Ion Monitoring (SIM) modes were performed under electron impact ionization mode at 70 eV in mass spectrometer. The quantification of compounds was done by internal standard method to monitor batch reproducibility and to correct for variations that occurred during sample preparation and analysis. 2-butanol was used as internal standard. Moreover, MSD ChemStation software (Agilent Technologies) was used to acquire mass spectrometric data. The mass spectra of all detected compounds were compared with spectra in the NIST library. The following ions were chosen for quantification: glycerol (61), acetol (74), acetone (58), acrolein (56), allyl alcohol (57), acetaldehyde (44) and acetic acid (60).
